# Patient Susceptibility to Candidiasis—A Potential for Adjunctive Immunotherapy

**DOI:** 10.3390/jof4010009

**Published:** 2018-01-09

**Authors:** Linda Davidson, Mihai G. Netea, Bart Jan Kullberg

**Affiliations:** Department of Internal Medicine and Radboud Center for Infectious diseases (RCI), Radboud University Medical Center, 6525 GA Nijmegen, The Netherlands; Linda.Davidson@radboudumc.nl (L.D.); Mihai.Netea@radboudumc.nl (M.G.N.)

**Keywords:** mucocutaneous candidiasis, invasive candidiasis, candidemia, immune defense, patient susceptibility, genetic predisposition, chronic mucocutaneous candidiasis (CMC), hyper IgE syndrome, immunotherapy

## Abstract

*Candida* spp. are colonizing fungi of human skin and mucosae of the gastrointestinal and genitourinary tract, present in 30–50% of healthy individuals in a population at any given moment. The host defense mechanisms prevent this commensal fungus from invading and causing disease. Loss of skin or mucosal barrier function, microbiome imbalances, or defects of immune defense mechanisms can lead to an increased susceptibility to severe mucocutaneous or invasive candidiasis. A comprehensive understanding of the immune defense against *Candida* is essential for developing adjunctive immunotherapy. The important role of underlying genetic susceptibility to *Candida* infections has become apparent over the years. In most patients, the cause of increased susceptibility to fungal infections is complex, based on a combination of immune regulation gene polymorphisms together with other non-genetic predisposing factors. Identification of patients with an underlying genetic predisposition could help determine which patients could benefit from prophylactic antifungal treatment or adjunctive immunotherapy. This review will provide an overview of patient susceptibility to mucocutaneous and invasive candidiasis and the potential for adjunctive immunotherapy.

## 1. Introduction

*Candida* spp., especially *Candida albicans*, are colonizing fungi of human skin and mucosae of the gastrointestinal and genitourinary tract of 30–50% of healthy individuals in any given human population at a certain moment, with the majority of the individuals being colonized during certain periods of their lives. Under normal conditions the human host defense prevents this commensal fungus from becoming a pathogen. Loss of skin or mucosal barrier function, microbiome imbalances, or defects of immune defense mechanisms can all lead to an increased susceptibility to severe mucocutaneous or invasive candidiasis [1].

Superficial candidiasis of mucosal membranes and skin is highly prevalent and occurs in immunocompromised as well as in apparently immunocompetent patients. In most cases these *Candida* infections of skin, nails, oropharyngeal mucosa, esophagus, and genital tract are occasional and not severe. Some patients, however, suffer from severe recurrent or persistent mucocutaneous *Candida* infections in the absence of commonly predisposing factors such as diabetes, and this condition has been termed chronic mucocutaneous candidiasis (CMC) [2].

Invasive candidiasis includes candidemia, deep-seated infections, and syndromes such as chronic disseminated (hepatosplenic) candidiasis. The mortality rate of invasive candidiasis is as high as 40%, which makes this a life-threatening condition. Known risk factors are indwelling vascular catheters, recent abdominal surgery, hematologic cancers, the administration of broad-spectrum antibiotic therapy, and multifocal *C. albicans* colonization. Interestingly, most patients admitted to the intensive care unit (ICU) have several of these risk factors but only a few develop invasive candidiasis [3].

Several genetic mutations and variations in immune regulation genes have been identified that confer susceptibility to *Candida* infections. Identification of patients with an underlying genetic predisposition could help determine which patients could benefit from prophylactic antifungal treatment or adjunctive immunotherapy [3,4]. This review will provide an overview of the mechanisms that increase patient susceptibility to mucocutaneous and invasive candidiasis and will provide a rationale of the potential for adjunctive immunotherapy.

## 2. The Host Immune Defense against *Candida*

### 2.1. Recognition of C. albicans

The first step of the host immune defence is recognition of an invading pathogen by the innate immune system. Recognition of microorganisms occurs through conserved chemical signatures, called pathogen-associated molecular patterns (PAMPs). These PAMPs are recognized by specific innate immune receptors known as pathogen recognition receptors (PRRs) [5,6]. The host is therefore able to orchestrate a pathogen-specific immune defense. The cell populations of the innate immune system involved in recognition of *C. albicans* are monocytes, macrophages, dendritic cells and neutrophils. These cells express PRRs in different patterns, rendering the host capable of a cell-type-specific immune defense against *C. albicans* [5].

The *Candida* cell wall consists of an inner layer of polysaccharides (chitin, 1,3-β-glucans and 1,6-β-glucans) and an outer layer of proteins glycosylated with mannan [1,5,6,7,8,9,10,11,12,13,14,15] constituting the PAMPs. The main PRRs that are involved in the recognition of *C. albicans* are the Toll-like receptors (TLRs) and the C-type lectin receptors (CLRs). TLR2 recognizes phospholipomannans, TLR4 recognizes *O*-linked mannans, the CLR dectin-1 recognizes β-glucans and the CLRs macrophage mannose receptor (MR) and dendritic cell (DC)-specific-ICAM3-grabbing non-integrin (DC-sign) recognize *N*-linked mannans [1,5,6,7,16,17,18,19,20,21,22,23,24,25,26,27]. The macrophage-inducible CLR (Mincle), expressed predominantly on macrophages, has been recognized as a receptor for *C. albicans* and plays a role in macrophage responses to *C. albicans* [28]. The ligand that binds to Mincle has not yet been identified. More recently, activation of the inflammasome through the nucleotide-binding oligomerization domain (NOD)-like receptor (NLR) pyrin domain-containing 3 (NLRP3) and the RigI-helicase receptor (RLR) melanoma differentiation-associated protein 5 (MDA5) has also been shown to be involved in anti-*Candida* host defense [29,30]. MDA5 is known for its recognition of viral RNA and its role in antiviral immunity, recently it has been suggested that MDA5 is also involved in antifungal immunity. Which ligands cause activation of MDA5 in *Candida* infection remains unclear [29,31].

**Figure 1:**
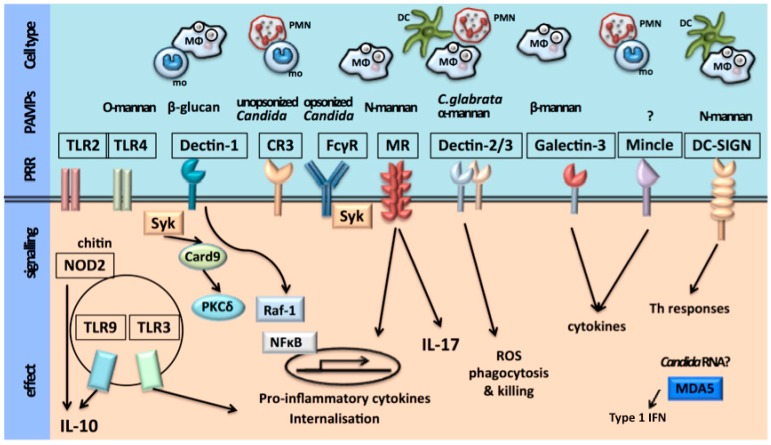
Recognition of *Candida* species by innate immune cells. Ligand binding to extracellular Toll-like receptors (TLRs), such as TLR2 and TLR4, leads to the production of pro-inflammatory cytokines during *Candida* infections. The intracellular TLRs that recognize nucleic acids—namely, TLR3 and TLR9—might also have a role in anti-*Candida* host responses. Chitin from *C. albicans* has been proposed to induce the production of interleukin-10 (IL-10) via a nucleotide-binding oligomerization domain-containing protein 2 (NOD2)-dependent mechanism and in this way may contribute to dampening pro-inflammatory host responses during fungal infection. The pattern recognition receptors (PRRs) dectin 1, dectin 2 and dectin 3, and Fc receptors for IgG (FcγRs), induce responses in a spleen tyrosine kinase (SYK)-dependent manner, whereas the signalling pathways engaged by the mannose receptor remain unknown. Dectin 1 can interact with TLR2 and can induce intracellular signalling via SYK- and RAF proto-oncogene serine/threonine-protein kinase (RAF1)-dependent pathways. Complement receptor 3 (CR3) is important for the recognition of unopsonized *Candida*, whereas FcγRs are important for recognition of opsonized *Candida* by neutrophils. Dendritic cell (DC)-specific -ICAM3-grabbing non-integrin (DC-SIGN) recognizes N-linked mannans of *Candida* and has a role in inducing T helper (TH) cell responses. There is no known *Candida*-derived ligand that triggers the macrophage-inducible C-type lectin receptor (MINCLE), whereas β-mannans from *Candida* are recognized by galectin 3. Although a role for melanoma differentiation-associated protein 5 (MDA5) in anti-*Candida* host responses has been described, it remains to be determined what ligand induces MDA5 activation. Together, these signalling pathways induce the secretion of cytokines and chemokines and initiate phagocytosis to clear *Candida* infections. CARD9, caspase activation and recruitment domain-containing 9; *C. glabrata*, *Candida glabrata*; NF-κB, nuclear factor-κB; PAMP, pathogen-associated molecular pattern; PKCδ, protein kinase Cδ; ROS, reactive oxygen species.

The recognition of *Candida* species by innate immune cells is depicted in Figure 1.

### 2.2. Activation of Host Immune Defense

Ligation of the PAMPs with their corresponding PRR leads to activation of the innate immune system, inducing production of pro-inflammatory cytokines and chemokines, and activation of the NLRP3 inflammasome, which processes pro-interleukin (IL)-1β and pro-IL-18 into their biologically active forms, with subsequent activation of inflammation and a directed adaptive immune response. Subsequently, these pathways potentiate phagocytosis and killing of *C. albicans*, predominantly by neutrophils [1,5,6,7,32,33,34,35,36,37,38,39,40,41,42,43,44,45].

Antigen-presenting dendritic cells are important for the activation of T cell responses. *Candida*-specific T helper cells (T*_H_*-cells) consist of T*_H_*1-cells and T*_H_*17-cells. T*_H_*1-cells are induced by IL-18 and produce interferon-gamma (IFN-γ). T*_H_*17-cells are induced by IL-1β and produce IL-17 and IL-22. IFN-γ is important for the fungicidal activity of neutrophils and macrophages. IL-17 and IL-22 induce neutrophil recruitment and activate neutrophils as well as epithelial cells and induce the release of antifungal β-defensins [1,5,6,46,47,48,49,50,51]. Activation via the intracellular PRR MDA5 induces a signaling pathway leading to the production of type I interferons. Recent studies have led to the suggestion that these interferons skew the adaptive cytokine response, induced by *C. albicans*, from a T*_H_*17 response to a T*_H_*1 response [29,31].

The composition of the host microbiome likely has significant impact on *Candida* colonization, invasion and host defense against *Candida*. As an example, the skin microbiome of patients with immunodeficiency disorders as CMC and hyper IgE syndrome (HIES) differs substantially from healthy individuals [52,53]. This topic will be discussed in another chapter of this special issue.

## 3. Mucocutaneous Candidiasis

Most cases of oropharyngeal and esophageal candidiasis occur in the setting of well-known risk factors, are incidental, and not severe. In these patients, use of broad-spectrum antibiotic therapy leads to *Candida* colonization on epithelial surfaces of skin, oropharynx, and vagina [54,55]. Development of vulvovaginal candidiasis following antibiotic therapy occurred in 20–22% of patients previously colonized with *Candida*. Studies show that only patients that were previously colonized with *Candida* had an increased risk of developing vulvovaginal candidiasis after antibiotic therapy [56,57]. Inhalation corticosteroids and systemic prednisone increase the risk of *Candida* infections [58]. Patients with diabetes have higher rates of vaginal *Candida* colonization and vulvovaginal candidiasis [59,60].

In addition, disruption of epithelial function, microbiome imbalances and loss of T cell function increases susceptibility to mucocutaneous candidiasis. Radiation therapy of the oro-maxillo-facial area leads to mucositis and all patients develop oral candidiasis at some point during therapy [61]. *Candida* infection in HIV patients is one of the so-called AIDS-defining illnesses. In absence of antiretroviral therapy, 57% of patients develop oropharyngeal candidiasis, typically when T cell CD4^+^ count is below 200 cells/m^3^ [62].

### 3.1. Epithelial Function

A major player in the host immune defense against mucocutaneous candidiasis is the epithelium and its interaction with *C. albicans*. The epithelium lining the oropharynx, skin and vagina forms the first physical barrier for invading pathogens. In addition to their physical barrier function, epithelial cells have an important immune function. Epithelial cells recognize *C. albicans* through the pattern recognition receptor (PRR) TLR4 and are able to differentiate between colonization and invasion. Recognition leads to the activation of the protein complex nucleair factor-kappaB (NF-κB) and the proto-oncogene c-Jun (JUN). Only when *Candida* germinates and forms hyphae invading the epithelium, a second response is initiated by activation of mitogen-activated protein kinase 1 (MAPK1) and FOS protein signaling. This triggers the initiation of the host response by releasing pro-inflammatory cytokines [6,7]. Recently, a fungal peptide toxin called Candidalysin has been discovered, secreted by *C. albicans* hyphae, that damages the epithelium and leads to immune activation of the epithelium by recognition of the peptide [63].

### 3.2. Th17 Pathway

The adaptive immune system plays a major role in the host defense against mucocutaneous candidiasis. Th17 cells, a differentiated subset of the CD4^+^ T-helper lineage, are induced after recognition of *C. albicans* mannan by the macrophage mannose receptor (MR). The dectin-1/TLR2 pathway enhances this response [64]. Th17 cells are activated via pro-inflammatory cytokines IL-1β, IL-6, TGF-β and IL-23 produced by antigen-presenting cells [65]. Production of IL-1β is predominantly induced by *Candida* hyphae via activation of the NLRP3 inflammasome and caspase-1. This means the Th17 pathway is only fully induced when *Candida* is invading, in contrast to colonizing mucosal tissue [66]. When activated, the Th17 cells produce IL-17A, IL-17F and IL-22. At early stages of infection, before induction of the Th17 subset, mucosa-associated type 3 innate lymphoid cells (ILCs) secrete IL-17 and thereby support host defense against mucocutaneous candidiasis [67].

IL-22 acts on epithelial cells to induce the release of antimicrobial peptides such as β-defensins. These β-defensins have potent fungicidal activity enabling the epithelium to prevent invasion of *C. albicans*. IL-17A and IL-17F induce neutrophil recruitment from the bloodstream to the site of infection where these cells aid to prevent *Candida* dissemination [6,7,68,69,70,71,72,73,74,75,76,77,78,79,80,81,82].

Induction of genes involved in neutrophil recruitment during oropharyngeal candidiasis is partly dependent on IL-17 receptor A activation [83]. Recently, it has been suggested that the role of IL-17 in neutrophil recruitment is dependent on the tissue environment [71]. In a mouse model of oropharyngeal candidiasis, the IL-17 pathway was not required for an adequate neutrophil response to *C. albicans* in the oral mucosa. Mice deficient in the IL-17 receptor A or depleted of IL-17A and IL-17F were able to establish normal neutrophil recruitment and function. Although the neutrophil response was normal, *Candida* colonization was persistent due to absent induction of epithelial antimicrobial peptides. These findings suggest that the main role of IL-17 in mucosal host defense against *Candida* is the stimulation of epithelial cells to release antimicrobial peptides, and to a lesser extent neutrophil recruitment [84]. Results from experimental psoriasis models in IL-17 receptor A deficient mice, support that neutrophil recruitment to the skin, is IL-17-independent [85].

Interestingly, while the Th17 pathway is important for defense against oropharyngeal candidiasis, it appears to be less important in the host defense against vulvovaginal candidiasis in mice [86]. Likewise, patients with genetic defects in their Th17 cell responses do not suffer from recurrent vulvovaginal candidiasis [2,87]. Major mechanisms involved in the pathogenesis of vulvovaginal candidiasis are local estrogen level, and imbalances in local microbiome [88]. In addition, IL-1β-induced hyperinflammation as a result of defects in the inflammasome seems to play a causal role in the pathogenesis of recurrent vulvovaginal candidiasis [30].

### 3.3. Genetic Susceptibility to Mucocutaneous Candidiasis

In the immunocompetent population, mucocutaneous candidiasis of skin, nails, oropharynx and esophagus occurs infrequent and is generally mild. Some patients, however, display a different clinical spectrum with severe and persistent *Candida* infections in the absence of risk factors. These syndromes comprise also the severe chronic mucocutaneous candidiasis (CMC) and hyper IgE syndrome (HIES). For most of these syndromes, the underlying genetic mutations or variation in immune regulation genes have now been identified.

#### 3.3.1. Chronic Mucocutaneous Candidiasis (CMC)

Patients with CMC suffer from severe and persisting *Candida* infections of skin, nails and mucous membranes. There are several CMC phenotypes caused by specific genetic defects.

Autoimmune polyendocrinopathy, candidiasis, and ectodermal dystrophy (APECED) was first described in 1929. This disorder is also known under the name autoimmune polyendocrine syndrome type 1 (APS-1). Mutations in the autoimmune regulator (*AIRE*) gene were found responsible in 1997, with an autosomal recessive transmission [1,89,90,91]. In the general population, APECED accounts for up to 20–40% of CMC cases, while it is the predominant form of CMC in Finns, Sardinians and Iranian Jews [92,93]. The clinical phenotype consists of CMC and autoimmunity, primarily of endocrine organs. The classic triad of CMC, hypoparathyroidism and adrenal insufficiency affects more than 80–90% of patients. Autoimmunity can also involve the gonads and thyroid and in small amounts of patients non-endocrine organs such as liver, eye, kidney and intestine [93]. The *AIRE* gene is expressed in the thymus and secondary lymphoid organs and plays a key role in immune tolerance. As part of autoimmune processes, all patients have autoantibodies against at least one out of three IL-17 cytokines (IL-17A (41%), IL-17F (75%) and/or IL-22 (91%)), which are essential for host defense against mucocutaneous *Candida* infection. Patients may also have auto-antibodies against IFN-α and IFN-ω, but do not display an increased susceptibility to viral infections [74,89].

In 2011, signal transducer and activator of transcription 1 (*STAT1*) mutations were discovered to likely be the main cause of autosomal dominant CMC [2]. STAT1 is a signaling molecule downstream of the type I and type II IFN receptors and of the IL-12 and IL-23 receptors. Gain-of-function mutations in the CC domain, the DNA-binding domain and the SH2 domain of *STAT1* cause disruption of the IL-12 and IL-23 pathways resulting in defective Th1 and Th17 cell responses and their consecutive production of IFN-γ, IL-17 and IL-22 [94]. Gain-of-function mutations of *STAT1* are associated with a broad clinical phenotype and are the most common genetic cause of CMC [95]. In addition to mucocutaneous candidiasis, 74% of patients with *STAT1* mutations suffer from cutaneous and respiratory bacterial infections (mainly *Staphylococcus aureus*), 38% suffer from viral skin infections (mainly Herpesviridae), 10% suffer from invasive fungal infections, and 6% suffer from mycobacterial infections [95]. Many patients with *STAT1* mutations also display autoimmune manifestations (37%), mainly hypothyroidism [95]. Esophageal carcinoma and cerebral aneurysms have also been linked to gain-of-function *STAT1* mutations [95,96]. The increased risk of invasive infections, squamous cell carcinoma and cerebral aneurysms make this disease potentially life-threatening.

Autosomal dominant *IL-17F*, autosomal recessive *IL17-RA*, autosomal recessive *IL-17RC* and autosomal recessive *TRAF3IP2* (encodes ACT1) gene mutations have also been reported to cause CMC in patients without mutations in the *AIRE* or *STAT1* genes. ACT1 is an adaptor molecule that interacts with the IL-17 receptor for downstream signaling response to IL-17A and IL-17F. Some of these patients also suffer from *S. aureus* skin infections [72,74,97].

Several gene mutations are associated with less severe manifestations of CMC. Autosomal recessive RAR Related Orphan Receptor C (*RORC*) gene mutations, causing RORγT deficiency affecting Th17 cell development, leads to mild CMC and severe mycobacterial infections. Autosomal recessive *IL-12B* or *IL-12Rβ1* gene mutations, causing IL-12Rβ1 or IL-12p40 deficiency, affecting both IL-12 and IL-23 signaling pathways, leads to mycobacterial infections in 83% of patients, to *Salmonella* infections in 43% and CMC in 23% of patients [74].

#### 3.3.2. Hyper IgE Syndrome (HIES)

HIES, also called Job syndrome, is characterized by recurrent staphylococcal skin abscesses, pulmonary aspergillosis, skeletal and dental abnormalities, eczema, eosinophilia, elevated serum immunoglobulin E concentrations, and mucocutaneous candidiasis [74].

Autosomal recessive inheritance of HIES is rare and caused mainly by mutations in dedicator of cytokinesis 8 (*DOCK8*) gene, encoding a protein involved in Th17 polarization [1,74]. Most cases of HIES are autosomal dominant and mainly caused by loss-of-function mutations in signal transducer and activator of transcription 3 (*STAT3*) [74]. STAT3 is a signaling molecule downstream of multiple cytokine receptors including IL-6, IL-10, IL-23, IL-17 and IL-22. The defective downstream signaling of the IL-23 receptor results in absent IL-17 production [74]. Of HIES patients with *STAT3* mutations, 85% develop CMC [1,74].

In addition to the typical skeletal and dental abnormalities, the clinical phenotype of HIES differs from CMC mainly by including allergic manifestations. Recent research shows that HIES patients are able to induce normal Th2 responses, while CMC patients have total absent Th2 responses [98].

#### 3.3.3. Recurrent Vulvovaginal Candidiasis (RVVC)

Vulvovaginal candidiasis is the most common form of mucocutaneous candidiasis in the immunocompetent host. A minority of women (5–8%) suffer from recurrent vulvovaginal candidiasis (RVVC), defined by recurrence of *Candida* infections more than three times a year, with many of these patients lacking any of the known clinical risk factors. Gene polymorphisms in PRRs, *TLR2* and mannose-binding lectin (*MBL*), and in the *NLRP3* inflammasome, and cytokine *IL-4*, have been discovered that play a role in the multifactorial susceptibility to RVVC.

The rs5743704 polymorphism in *TLR2*, increased the susceptibility to RVVC almost three-fold, in a study with 119 RVVC patients, functional assays suggested that this polymorphism reduces production of IL-17 and IFN-γ upon stimulation of peripheral blood mononuclear cells with *C. albicans* [99]. Polymorphisms in codon 54 in *MBL2*, has been linked to RVVC. This *MBL2* gene codes for the soluble PRR mannose-binding lectin (MBL) which promotes complement activation and *Candida* killing [100]. In a group of 50 women with RVVC, 25% carried this polymorphism compared to 10.6% in controls [101].

The rs74163773 polymorphism in the *NLRP3* inflammasome is associated with an increased susceptibility to RVVC, as studied in a group of 270 RVVC patients. This polymorphism leads to hyperinflammation by overproduction of IL-1β. Levels of IL-1β at the vaginal surface were higher in patients bearing this polymorphism, IL-1Ra levels were decreased [30].

The −589C/T polymorphism in the *IL-4* gene is associated with an increased susceptibility to RVCC, in a group of 42 women with RVVC 59.5% had this polymorphism compared to 7.0% in controls. This polymorphism leads to an elevated concentration of IL-4 and decreased concentration of MBL and nitric oxide (NO) in vaginal fluid. IL-4 has been known to inhibit macrophage activation and NO production [102].

A very recent study has performed the first GWAS in patients with RVVC. In this study, the most important pathways regulating susceptibility to RVVC at a genetic level have been revealed, including cytokine production capacity, cellular morphogenesis and metabolism, as well as cell adhesion (Jaeger et al., personal communication).

#### 3.3.4. *Candida* Colonization, Cutaneous Candidiasis and Onychomycosis

The Tyr238X polymorphism in an early stop codon in the *dectin-1* gene, increases susceptibility to oral and gastrointestinal *Candida* colonization and onychomycosis, by defective β-glucan recognition and consecutive Th17 cell responses. Screening for *dectin-1* polymorphism in patients undergoing hematopoietic stem cell transplantation, showed 10.6% of patients bearing this polymorphism, they were significantly more often colonized with *Candida* [103]. This polymorphism is present in up to 8% of Europeans and up to 40% of selected sub-Saharan African populations [103,104].

The L412F polymorphism in *TLR3*, is associated with chronic cutaneous candidiasis in patients without known genetic mutations conferring susceptibility to CMC [105]. Peripheral blood mononuclear cells of patients carrying this polymorphism, showed reduced IFN-γ and tumor necrosis factor alpha (TNF-α) secretion on response to stimulation with cytomegalovirus (CMV) and *C. albicans.* These patients seem to have an increased risk of CMV infection and autoimmune manifestations as well.

## 4. Invasive Candidiasis

Imbalances in microbiome caused by antibacterial agents, disruption of the barrier between the external and internal environment and loss of neutrophil function are well known risk factors for invasive candidiasis. Patients admitted to the ICU acquire *Candida* colonization in up to 80% of patients during the first 7 days [106]. Multifocal *Candida* colonization in combination with abdominal surgery or indwelling vascular catheters are major risk factors for the development of invasive candidiasis [3]. Of candidemia episodes, approximately 50% occur in the ICU, with 70–90% of patients having previously been exposed to broad-spectrum antibiotic therapy, 80–90% have an intravascular device in situ at the time of candidemia, and 40–50% have had recent surgery [107,108,109]. Remarkably, neutropenia is only present in less than 5% of patients with candidemia [108,109].

*Candida* colonizing the gut invades either through translocation, or through anastomotic leakage after laparotomy, and may cause localized, deep-seated infection (e.g., peritonitis), or candidemia via the portal circulation [3]. During candidemia, dissemination of *Candida* may lead to secondary metastatic lesions, e.g., in lung, liver, spleen, kidneys, bone and eye. Also indwelling intravascular catheters are likely to become colonized [3].

Patients with hematologic malignancies receiving anticancer chemotherapy may develop extensive mucositis of the gastrointestinal tract, and hence be at risk for translocation of colonizing *Candida* to the blood stream. In patients with prolonged neutropenia, chronic disseminated candidiasis is a rare specific entity [110]. This form of invasive candidiasis primarily involves the liver and spleen, and often only becomes overt after neutrophil recovery. Immune reconstitution inflammatory syndrome (IRIS) plays a major part in the disease pathogenesis [110].

### 4.1. Th1 Pathway and Neutrophil Function

Th1 cell responses are crucial for protection against invasive candidiasis, and IFN-γ is the pivotal cytokine in anti-*Candida* host defense. The pro-inflammatory cytokine IL-18, processed by the inflammasome, induces Th1 cell responses such as IFN-γ production [43]. IFN-γ has a stimulating effect on the fungicidal activities of phagocytic cells mainly neutrophils and macrophages [48,49]. Natural killer cells (NK) further enhance this fungicidal activity by producing granulocyte -macrophage colony-stimulating factor (GM-CSF).

Neutrophil activation is essential for the clearance of *Candida*, as these are the most potent phagocytes to kill *Candida*, and the only host immune cell able to inhibit germination of yeasts into hyphae. After phagocytosis of *Candida*, neutrophils use oxidative as well as non-oxidative killing mechanisms. The production of reactive oxygen species (ROS), is mediated through a protein complex called nicotinamide adenine dinucleotide phosphate (NADPH) oxidase. The enzyme myeloperoxidase (MPO) catalyses the conversion of hydrogen peroxide to hypohalous acid, which amplifies the toxicity of ROS. The fungicidal activity relies on toxicity of ROS and release of antifungal proteases. This ROS-dependent mechanism is essential for clearance of opsonized *Candida*, and depends on binding to the FcγRs receptor and protein kinase C activation. Non-oxidative killing proceeds by producing anti-microbial factors such as lysozyme, lactoferrin, elastase, β-defensins, gelatinases and cathepsin G. In addition to killing by phagocytosis, neutrophils can release chromatin fibers forming neutrophil extracellular traps (NETs). These NETS are able to kill *Candida* yeasts as well as hyphae, by binding them and releasing an antifungal peptide called calprotectin. This ROS-independent mechanism is essential for clearance of unopsonized *Candida*, and depends on binding to the CR3 receptor and CARD9 activation [6,7,111,112].

### 4.2. Genetic Susceptibility to Invasive Candidiasis

Even among patients with a combination of several risk factors for invasive candidiasis, the disease occurs in only a minority of patients. This suggests a role for underlying genetic variations, which, in combination with several risk factors, makes a patient prone to develop invasive candidiasis. Several polymorphisms in immune regulation genes have recently been described, leading to either an increased susceptibility to acquire or a decreased ability to clear invasive candidiasis.

#### 4.2.1. Increased Susceptibility to Acquire Candidemia

The influence of polymorphisms in TLRs genes on susceptibility to candidemia was prospectively studied in a large case and control cohort consisting of European and North American hospitalized patients at risk for candidemia [113]. An association with increased susceptibility to acquiring candidemia was found for three polymorphisms in *TLR1* gene. Patients with these genotypes display decreased proinflammatory cytokine release upon stimulation ex vivo. No association was found for polymorphisms in *TLR2*, *TLR4*, *TLR6*, *TLR9*, or their adaptor proteins myeloid differentiation primary response 88 (*MyD88*) or TIR domain containing adaptor protein (*TIRAP*) genes [113]. Three additional polymorphisms in *CD58*, *LCE4A-C1orf68*, and T-cell activation GTPase activating protein (*TAGAP*) loci associated with increased susceptibility to candidemia were identified in a genome-wide association study (GWAS) in the largest candidemia cohort to date. The risk of candidemia for patients on the ICU was increased by more than 19-fold when carrying at least two risk alleles from these loci. *CD58* is important for *Candida* phagocytosis and inhibition of germination, *TAGAP* has a function in *Candida*-induced cytokine production, and *LCE4A-C1orf68* locus contributes to mucosal integrity [114]. Recently, 18 novel susceptibility loci were validated, by integrating genotype data from a cohort of 217 candidemia patients with transcriptome changes in healthy human primary leucocytes induced by *Candida*. In these 18 loci, 31 candidate genes were identified [115].

#### 4.2.2. Increased Susceptibility to Persistent Candidemia

Persistent candidemia is defined as positive blood cultures yielding *Candida* for more than five days despite adequate therapy. In a large prospective cohort of ICU patients with candidemia, polymorphisms in cytokine genes encoding IL-10 and IL12B were found to be associated with persistent candidemia [116]. This decreased capability to clear the blood stream from *Candida* is likely mediated by increased production of the anti-inflammatory cytokine IL-10 and decreased production of IL-12b, resulting in downregulation of IFN-γ production. Polymorphisms in *IFN-γ*, *IL-18*, *IL-1β* and *IL-8* genes were not associated with persistent candidemia [116]. In a subsequent GWAS study, the *CD58* polymorphism was also associated with persistent candidemia [114].

#### 4.2.3. Increased Susceptibility to *Candida* Dissemination

Disseminated candidiasis is defined as the presence of *Candida* at normally sterile sites outside the bloodstream, and this condition may be either acute or chronic.

An association between genetic variants in *IL-4* gene and susceptibility to chronic disseminated candidiasis (CDC) was found in a cohort of 90 patients with acute leukemia, of which 40 suffered from CDC [117]. The −1098T/−589C/−33C polymorphism in *IL-4* gene was associated with increased susceptibility to CDC in acute leukemia patients whereas the −1098T/−589T/−33T polymorphism was associated with decreased susceptibility. IL-4 is an anti-inflammatory cytokine, induced by Th2 cell responses. The polymorphism that increased susceptibility to CDC was associated with a decreased IL-4 transcriptional activity [117].

The autosomal recessive inheritance of loss-of-function mutations in caspase recruitment domain-containing protein 9 (*CARD9*) gene, were first described in a large consanguineous Iranian family with recurrent mucocutaneous and invasive *Candida* infections [118]. Interestingly, this is the first, and as yet only, described genetic cause for a combined phenotype of mucocutaneous and invasive candidiasis. CARD9 is an intracellular adaptor molecule essential for dectin-1 signaling. This signaling pathway induces the production of IL-1β, IL-6, and IL-23, and consecutive Th17 cell responses. In the four affected family members, low numbers of circulating Th17 cells were found. Three affected family members died during adolescence, two died from *Candida* meningoencephalitis, and one died from presumed, but not confirmed, *Candida* cerebral involvement [118].

In a subsequent study, additional understanding was obtained on the invasive nature of candidiasis resulting from mutations in *CARD9* gene [119]. Neutrophils of a patient diagnosed with *Candida* meningoencephalitis and *CARD9* gene mutations, displayed a selective *Candida* killing defect, which was independent of ROS production. The underlying mechanism is not yet fully understood [119]. In five additional patients, with *CARD9* gene mutations, who all were born to consanguineous parents of Arabic origin, an association of *CARD9* gene mutations with tissue culture proven *Candida* colitis was described [120]. These observations support the role for CARD9 in anti-fungal immunity of the gut, as was previously found in murine models. CARD9-deficient mice displayed strong fungal colonization of the digestive tract with decreased numbers of colonic Th17 cells and innate lymphoid cells [120]. The earlier mentioned polymorphism in *TAGAP* gene is also associated with dissemination in organs [114]. An overview of genes involved in genetic susceptibility to *Candida* infections is depicted in Table 1. 

## 5. Translating Knowledge into Clinical Practice

Morbidity and mortality associated with *Candida* disease remains substantial, despite advances in supportive care and novel antifungal agents, demanding improvement of antifungal therapy. The current knowledge on pattern recognition, Th1 and Th17 cell responses, and host effector mechanisms in candidiasis underlines the potential for adjunctive immunotherapy in antifungal treatment. Identifying patients within high-risk groups who bear genetic mutations or polymorphisms associated with specific immune pathway defects allows selection of subjects for whom antifungal prophylactic therapy, early empiric treatment, or host-directed adjunctive immunotherapy is expected to be most effective. Thus, more accurate definition of high-risk groups will, in light of overtreatment and emergence of fungal resistance, provide an additional benefit on a population level [3,121].

### 5.1. Prophylaxis

Antifungal prophylaxis on ICU is currently used only in specific high-risk groups, in which it has been shown to be effective, such as abdominal surgery with recurrent gastrointestinal anastomotic leakage, transplantation of the pancreas or small bowel, liver transplantation in selected patients who are at high risk of candidiasis, and extremely low-birth-weight neonates in settings with a high incidence of neonatal candidiasis [122].

Benefit of antifungal prophylaxis can, however, be expected in other ICU patient groups with additional risk factors, such as broad-spectrum antibiotic therapy use, gastrointestinal disease and indwelling vascular catheters. Studies to evaluate the value of antifungal prophylactic therapy have been based on defining these high-risk groups by clinical parameters. However, a recent randomized, placebo-controlled study in which patients admitted to ICU with a high risk of acquiring invasive candidiasis based on a clinical prediction rule, received antifungal prophylaxis, yielded no significant difference in candidemia rate or overall mortality [123]. Screening for genetic mutations and polymorphisms in immune regulation genes within these high-risk patient groups, may improve risk stratification to determine which patients need antifungal prophylaxis.

### 5.2. Immunostimulatory Therapy

#### 5.2.1. Recombinant Cytokine Therapy

To date, recombinant cytokine therapy is the only clinically available form of adjunctive antifungal immunotherapy and relies on improving host immune effector functions. Colony-stimulating factors have been considered as antifungal adjunctive therapy [121,124,125]. These factors enhance phagocytosis and the release of ROS, prolong the survival of neutrophils by inhibiting programmed cell death and upregulate chitotriosidase promoting fungicidal activity. In addition, GM-CSF is known to stimulate upregulation of dectin-1 expression on macrophages. GM-CSF has been successfully used in HIV patients with refractory mucosal candidiasis [126,127]. No studies with GM-CSF have been performed in patients with invasive candidiasis. Granulocyte colony-stimulating factor (G-CSF) was shown to be effective in mice with disseminated candidiasis [128]. In a randomized placebo-controlled pilot study among non-neutropenic patients with disseminated candidiasis, adjunctive immunotherapy with G-CSF in combination with fluconazole showed a trend towards faster resolution of infection than fluconazole alone [129]. No follow-up studies have been done to date.

IFN-γ has been shown to enhance candidacidal activity of macrophages and neutrophils. Administration of IFN-γ to mice with disseminated candidiasis reduces fungal burden [130]. In an open-label prospective pilot study, patients with candidemia have received adjunctive IFN-γ or placebo. Adjunctive IFN-γ immunotherapy partially restored antifungal immune function in the setting of sepsis-induced immune paralysis, with improvement in leukocyte innate immune responses (IL-1β, TNFα), and increased production of T-lymphocyte cytokines (IL-17 and IL-22). This study was not powered to show effect on mortality [131].

#### 5.2.2. Vaccination and Antibodies

Passive immunization with anti-*Candida* antibodies could be of benefit in immunocompetent as well as immunodeficient patients. Screening among candidiasis patients for polymorphisms known to decrease the ability of *Candida* clearance, such as the polymorphism in *IL-10* and *IL-12B* genes, may guide selection of patients most likely to benefit from adjunctive antibody therapy. Protective monoclonal antibodies against *Candida* have been developed and tested successful in murine models [124,132]. Anti-*Candida* antibodies are not clinically available yet.

Active vaccination for candidiasis would be the ultimate prevention method in immunocompetent patients undergoing elective procedures or treatments known to increase the risk of candidiasis. Extensive research over the years has provided several potential vaccines but none of these is clinically available yet. Two vaccines, containing recombinant *C. albicans*-derived proteins, have reached phase II trials. The NDV-3 vaccine, a recombinant alum-adjuvanted vaccine for *Candida* and *Staphylococcus aureus*, has been shown to protect mice against oropharyngeal, vulvovaginal, and invasive candidiasis as well as skin and soft tissue infections with *S. aureus* [133]. In phase I trials it has shown promising results in T en B cell responses [134]. A memory T-cell response was shown for IFN-γ in almost all participants, and for IL-17 in the majority of participants. The SAP2 vaccine, studied for its effect on vulvovaginal candidiasis, has shown to generate neutralizing vaginal antibodies [4,121]. Interestingly, studies have shown that by adding different adjuvants to vaccines, different cytokine profiles and Th cell responses are induced. By adding an adjuvant specific for mucocutaneous disease or for invasive disease, a vaccine may be expected to shape the adaptive immune response towards either a Th17 or a Th1 response [124].

#### 5.2.3. Innate Cellular Immunotherapy

Granulocyte transfusion, as treatment for sepsis in neutropenic patients, was developed in the 1970s. Toxicity of the transfusion, development of haematologic growth factors, and antimicrobial and antimycotical agents have halted its use. The availability of recombinant GM-CSF and G-CSF, however, has inspired new trials based on the expected higher yield of donor granulocytes. No difference in survival was found in a randomized phase III trial in febrile neutropenia patients with fungal disease, receiving granulocyte transfusion adjunctive to standard of care [135]. Similarly, no difference in the overall success between adjunctive granulocyte transfusion or antibiotic therapy alone was found in a multicentre randomized trial in patients with febrile neutropenia and presumed infection [136]. In conclusion, the clinical efficacy of granulocyte transfusion has never been conclusively demonstrated. Another potential option for antifungal immunotherapy, yet to be studied in man, is dendritic cell-vaccination, in which dendritic cells are primed ex vivo with antigens that induce specific cytokine profiles that induce an anti-*Candida* host response, and are then infused in patients with candidiasis [121,124].

### 5.3. Immunosuppressive Therapy

In specific types of candidiasis, suppressing, rather than stimulating, host antifungal immune response may be beneficial. In chronic disseminated candidiasis, a hyperactive immune response occurs when neutrophil count returns to normal (IRIS). Adjunctive corticosteroid therapy, for at least three weeks up to one year, has been described to lead to resolution of symptoms and of inflammatory response [137]. Anakinra, a recombinant IL-1Ra and potent suppressor of inflammasome activity, has proven effective in mice studies of VVC [138]. Thus, based on the hyperinflammation as result of defects in the NLRP3 inflammasome and successive increase in IL-1β production in RVVC patients, a potential role for anakinra as antifungal immunotherapy has been suggested.

### 5.4. Future Perspectives

Increased knowledge about host immune response to *Candida* infections, and genetic mutations and variations in immune regulation genes conferring susceptibility to candidiasis has led to development of various forms of immunotherapy that show potential as adjunctive antifungal therapy. New development of immunotherapy could comprise recombinant cytokine IL-17 or IL-22, considering their value in antifungal immune responses.

Prospective studies are now warranted to investigate the efficacy of including genetic screening, within high-risk patient groups, in stratifying patient risk for candidiasis, and to evaluate the efficacy of antifungal prophylaxis and adjunctive antifungal host-directed immunotherapy when administered in this selection of patients.

## Figures and Tables

**Table 1:** Genes involved in genetic susceptibility to *Candida* infections.

Disease	Gene	Immune Modification	Infectious Phenotype	Non-Infectious Phenotype	References
CMC	*AIRE*	Autoantibodies against at least one out of three IL-17 cytokines; IL-17A (41%), IL-17F (75%) and/or IL-22 (91%)	CMC	Autoimmune manifestations: hypoparathyroidism and adrenal insufficiency	[89,90,91,92,93]
	*STAT1* (gain-of-function)	Disruption of the IL-12 and IL-23 pathways resulting in defective Th1 and Th17 cell responses and their consecutive production of IFN-γ, IL-17 and IL-22	CMC, cutaneous and respiratory bacterial infections (mainly *Staphylococcus aureus*), viral skin infections (mainly Herpesviridae), invasive fungal infections	Autoimmune manifestations (hypothyroidism, autoimmune hemolytic anemia, etc), esophageal carcinoma, cerebral aneurysms	[2,94,95,96]
	*IL-17RA*, *IL-17F, IL-17RC*, *TRAF3IP2* (encodes ACT1)	Deficiency of IL-17RA, IL-17F , IL-17RC, ACT1 causing disruption of the downstream signaling response to IL-17A and IL-17F	CMC and *S. aureus* skin infections	-	[72,74,97]
	*RORC*	RORγT deficiency affecting Th17 cell development	Mild CMC and severe mycobacterial infections	-	[74]
	*IL-12B* or *IL-12Rβ1*	IL-12Rβ1 or IL-12p40 deficiency, affecting both IL-12 and IL-23 signaling pathways	CMC, mycobacterial infections, and *Salmonella* infections	-	[74]
HIES	*STAT3*	Defective downstream signaling of the IL-23 receptor resulting in absent IL-17 production	Mucocutaneous candidiasis, recurrent staphylococcal skin abscesses and pulmonary aspergillosis	Skeletal and dental abnormalities, pneumatoceles, eczema, eosinophilia, and elevated serum immunoglobulin E concentrations	[74]
	*DOCK8*	Disruption in Th17 differentiation	Mucocutaneous candidiasis, recurrent staphylococcal skin abscesses and pulmonary aspergillosis	Eczema, eosinophilia and, elevated serum immunoglobulin E concentrations	[1,74]
RVVC	*TLR2*	Reduced production of IL-17 and IFN-γ	RVVC	-	[99]
	*IL-4*	Elevated concentration of IL-4 and decreases concentration of MBL and nitric oxide (NO) in vaginal fluid	RVVC	-	[102]
	*MBL2*	Reduced complement activation and *Candida* killing	RVVC	-	[100]
	*NLPR3*	Hyper-inflammation by overproduction of IL-1β, high levels of IL-1β and low levels of IL-1Ra at the vaginal surface	RVVC	-	[30]
Onychomycosis	*Dectin-1*	Defective β-glucan recognition and consecutive Th17 cell responses	Onychomycosis and *Candida* colonization oral and gastrointestinal	-	[103,104]
Cutaneous candidiasis	*TLR3*	Reduced CMV and *Candida*-induced IFN-γ and TNF-α production	Cutaneous candidiasis and CMV infection	Autoimmune manifestations: hypothyroidism, hypogonadism, idiopathic thrombocytopenic purpura, pancytopenia, alopecia, enteritis	[105]
Candidemia	*IL-10*	Increased production of the anti-inflammatory cytokine IL-10	Increased susceptibility to persistent candidemia	-	[116]
	*IL-12B*	Decreased production of the pro-inflammatory cytokine IL-12b, resulting in downregulation of IFN-γ production.	Increased susceptibility to persistent candidemia	-	[116]
	*TLR1*	Decreased *Candida*-induced cytokine production	Increased susceptibility to acquire candidemia	-	[113]
	*CD58*	Disruption of *Candida* phagocytosis and loss of inhibition of germination	Increased susceptibility to acquire candidemia	-	[114]
	*LCE4A-C1orf68*	Disruption of mucosal integrity	Increased susceptibility to acquire candidemia	-	[114]
	*TAGAP*	Decreased *Candida*-induced cytokine production	Increased susceptibility to acquire candidemia	-	[114]
CDC	*CARD9*	Low numbers of ciruculating Th17 cells and ROS-independent selective *Candida* killing effect neutrophils	Recurrent mucocutaneous and invasive *Candida* infections (meningoencephalitis, colitis)	-	[118,119,120]
	*IL-4*	Decreased IL-4 transcriptional activity	Increased susceptibility to CDC in acute leukemia patients	-	[117]
	*TAGAP*	Decreased *Candida*-induced cytokine production	Increased susceptibility to *Candida* dissemination into organs	-	[114]

CMC: chronic mucocutaneous candidiasis; HIES: hyper IgE syndrome; RVVC: recurrent vulvovaginal candidiasis; CDC: chronic disseminated candidiasis
